# Injection Molding of Low-Density Polyethylene (LDPE) as a Model Polymer: Effect of Molding Parameters on the Microstructure and Crystallinity

**DOI:** 10.3390/polym13203597

**Published:** 2021-10-19

**Authors:** César Leyva-Porras, Andrea Balderrama-Aguilar, Yael Estrada-Ávila, Iñaki Espelosín-Gómez, Mónica Mendoza-Duarte, Claudia Piñón-Balderrama, María Zenaida Saavedra-Leos, Iván Estrada-Moreno

**Affiliations:** 1 Centro de Investigación en Materiales Avanzados S.C. (CIMAV), Complejo Industrial Chihuahua, Miguel de Cervantes No. 120, Chihuahua 31136, Mexico; monica.mendoza@cimav.edu.mx; 2 Tecnológico Nacional de México, Campus Chihuahua (ITCH), Av. Tecnológico No. 2009, Chihuahua 31310, Mexico; 6f.balderrama.aguilar.andrea@gmail.com (A.B.-A.); yael982010@gmail.com (Y.E.-Á.); iniakyesgo@gmail.com (I.E.-G.); 3 Departamento de Ingeniería Industrial, Universidad Tecnológica de Chihuahua (UTCH), Montes Americanos No. 9501, Chihuahua 31216, Mexico; claudia.pinon@cimav.edu.mx; 4 Coordinación Académica Región Altiplano, Universidad Autónoma de San Luis Potosí, Carretera Cedral Km. 5+600 Ejido San José de las Trojes, Matehuala 78700, San Luis Potosí, Mexico; zenaida.saavedra@uaslp.mx

**Keywords:** injection molding, low-density polyethylene (LDPE), microstructure, spherulites, ANOVA, response surface methodology

## Abstract

Due to its relatively simple structure, low-density polyethylene (LDPE) can be considered as a model polymer for the study of its properties. Herein, the effect of processing variables on the microstructure and crystallinity of injection-molded LDPE specimens was quantitatively determined. The polymer was injected at different temperature conditions in the barrel and the mold. The specimens were characterized by scanning electron microscopy and X-ray diffraction. With the data obtained, an analysis of variance (ANOVA) was carried out, and response surface graphs (SRP) were constructed to quantify and to observe the behavior of the processing variables, respectively. Different models were obtained to predict the effect of the experimental factors on the response variables. The results showed that the interaction of the two temperatures has the greatest effect on the size of the spherulite, while the temperature of the mold affects the crystallinity. The SRP showed different behaviors: for the spherulite, the size increases with the mold temperature, while for the crystallinity, higher values were observed at an intermediate mold temperature and a low melt temperature. The results presented herein are valuable for setting empirical relations between the microstructure, crystallinity, and the molding conditions of LDPE.

## 1. Introduction

Polyethylene (PE) is a commodity polymer widely used in the plastics market, occupying about one-third of the total production in the world [1]. Based on the complex structural hierarchy, there are three different types of PE, named low-density PE (LDPE), high-density PE (HDPE), and linear low-density PE (LLDPE), with all of these polymers differing in molecular weight distribution and chain branching [2,3]. LDPE is employed in the manufacturing of bottles, films, wire insulators, and molded parts [4,5,6,7]. In the academic field, it is used as a model polymer for the understanding of the relation between the structure and properties [1,8,9]. Although LDPE exhibits a spherulitic supramolecular structure in the bulk of thick specimens (>200 nm) such as those injected molded, other morphologies may be obtained by varying the film thickness in confined systems [10,11,12]. Because of its semicrystalline nature, the microstructure presents ordered regions of polymer chains known as spherulites, which are conformed of fold back chains or lamellae growing out from nucleation points, interconnected by disordered polymer chains or amorphous regions [6]. The ratio of the crystalline to amorphous regions influences the different physical properties, such as mechanical, thermal, surface, and hardness [13,14]. Consequently, several works have qualitatively related this ratio with the final measured properties [10,15,16,17,18,19,20,21,22]. Zheng et al. [6] employed X-ray diffraction (XRD) to calculate the intensities ratio of the amorphous halo to the most intense diffraction peak of LDPE hot-pressed at different conditions. They found different results when comparing the calculated ratios against the full-width half-maximum (FWHM) values. Alapati, Meledath, and Karmarkar [5] employed differential scanning calorimetry (DSC) to determine the melting temperature (T_m_) and crystallinity degree of LDPE filled with alumina nanoparticles. They observed that T_m_ remained unchanged while the crystallinity decreased by about 3% in the filled polymer. Nilsson, Hjertberg, and Smedberg [10] studied the effect of the crosslinking degree on the size and shape of the supramolecular structure of LDPE. They explained that crosslinking decreases the size of the spherulite and modifies its shape. Ma et al. [18] showed that the addition of TiO_2_ nanoparticles did not affect the overall crystallinity of LDPE but disordered the lamellar organization. Wang et al. [21] demonstrated by XRD and DSC that, even though polyethylene terephthalate (PET) may act as a nucleating site for the crystallization of LDPE, the addition of recycled PET did not affect the crystal form of LDPE. Picu and Osta [20] determined the elastic modulus by AFM indentation of isotactic polypropylene (iPP) spun fibers cooled at a high cooling rate (>100 °C/min). They found that the modulus was 52% lower when measured in the axial direction of the fiber than that measured in the transversal direction. Kalay et al. [23] employed XRD and DSC techniques to evaluate changes in the crystallinity of HDPE samples. Their results showed an increment in the intensity of the diffraction peak and a larger enthalpy of fusion in the shear-controlled oriented samples.

On the other hand, the development of quantitative relations requires the measurement of the spherulite size and the use of statistical tools to relate the size measurements with the properties. In this sense, the observation of spherulites in thick samples is not a straightforward task but involves adequate sample preparation, including polishing, chemical etching, and contrasting with staining agents [24]. The most common observation methods of spherulites include light microscopy, birefringence studies, amplitude-modulation atomic force microscopy (AM-AFM), scanning electron microscopy (SEM), and transmission electron microscopy (TEM) [25,26,27]. The analysis of variance (ANOVA) is a statistical tool generally employed to quantify the contribution of the experimental factors on the response variables and their interactions and calculate the experimental error within a significance value [8,28,29,30]. Although several works have used ANOVA in the optimization of the processing conditions of LDPE, HDPE, or polypropylene (PP) [23,31,32,33,34,35], only a few have related the processing conditions with the size of the spherulites [36,37,38,39]. For example, Katti and Schultz [33] reviewed the microstructure of injection-molded semicrystalline polymers, including polyethylene. They identified four distinct morphological zones in cross-section samples that showed differences in the locally measured properties, such as yield stress. Guo, Isayev, and Demiray [37] carried out a complete study of the effect of injection molding conditions on the spherulite’s size of iPP, finding that mold temperature was the variable with the largest effect on the increment of the size of the spherulites. Almanza et al. [35] found that the size and morphology of the spherulites of LDPE foams are different from those of thin sheets, and consequently, the macroscopic properties, such as Young’s modulus. Recently, Leyva-Porras et al. [8] developed an empirical approach derived from the ANOVA and surface response methodology (SRM) to predict the Young’s modulus of LDPE processed at different mold and barrel temperature conditions of injection-molded specimens. Among their findings, it was reported that mold temperature was the main variable affecting the Young’s modulus, and qualitatively, they discussed the microstructure changes in terms of the degree of crystallinity. Therefore, it is still necessary to establish empirical relationships based on phenomenological observations that can quantitatively associate the microstructure with the macroscopic properties.

In this sense, the present work aims to establish quantitative relationships between the mold and barrel temperatures for the injection molding process of LDPE specimens. For this purpose, an experimental design of type 3^2^ was performed, and the obtained specimens were analyzed. ANOVA and SRM were carried out for each of the characterizations, including the size of the spherulites and the crystallinity degree. The work contributes to understanding the way the microstructure of LDPE varies with the injection-molding processing conditions and its relation to the macroscopic properties, such as crystallinity.

## 2. Materials and Methods

### 2.1. Materials

Low-density polyethylene (LDPE) thermoplastic resin (product No. PX-18450 G, injection grade, PEMEX, Mexico City, Mexico) with a flow index of 45 g/10 min and density of 0.9185 g/cm^3^ was employed as raw material for the injection molding of the specimens. Thermal properties of LDPE presented a melting enthalpy of 42.8 J/g and a melting temperature of 107.1 °C. Pellets were dried overnight at 80 °C to remove any surface moisture.

### 2.2. Design of Experiments

A factorial design allows determining the effect of a given factor at various levels on one or more response variables [28]. A full factorial experimental design of type 3^2^ with 10 replicates was used. The two factors tested were the melt temperature and mold temperature; the three levels for each of the factors were varied as 120, 140, and 160 °C for melt temperature, and 10, 40, and 80 °C for mold temperature. The combination of these factors and the corresponding levels results in a set of nine experiments.

The temperature profile inside the injection barrel consisted of four zones identified as feeding zone (T_F_), compression zone (T_P_), dosing zone (T_D_), and nozzle zone (T_N_). The temperature variation between the zones was 5 °C, where T_F_ < T_D_ < T_P_ < T_N_. The melt temperature reported herein corresponds to the injection nozzle zone. The temperature range used in this work was selected based on the previously reported results by Leyva-Porras et al. [8], who observed a greater variation in the results in the lower temperature range.

### 2.3. Injection Molding of the Specimens

The injection molding of the specimens was carried out in an industrial injection-molding machine (Negri-Bossi, model V55-200, Milan, Italy). Mold cavities met the sample dimensions specified in ASTM D 638. For example, the dimensions of the necked specimens were 25.4 × 2.54 × 0.254 cm (L × W × T). Melt and mold temperatures were varied as described in the design of experiments section. The injection-molding settings, such as molding pressure, holding pressure, injection volume, cooling time, among others, were kept constant during all the experiments; these settings are described in Table 1. For each experiment, fifteen injection-molding cycles were carried out, removing the first five samples and storing the last ten.

### 2.4. Morphological and Microstructural Characterization

XRD was employed for analyzing the overall crystallinity of the molded specimens. Analyses were carried out in with a Malvern Panalytical Empyrean X-ray diffractometer (Panalytical B.V., Almelo, The Netherlands) equipped with Cu-k_α_ radiation and θ−2θ geometry in the range of 15−70 2θ degrees, step size of 0.02°, and 3 s per step. A complete specimen was introduced in the diffractometer sample holder, analyzing the central region of the specimen with an area of approximately 2 × 2 cm. Three zones on each specimen were analyzed.

For observing the spherulites in the bulk of LDPE molded specimens, several micrographs were acquired using a scanning electron microscope (SEM) Hitachi SU3500 (Tokyo, Japan) operated at 15 kV, low vacuum conditions (60 Pa), and with a backscattered electron detector (BSE). Micrographs were acquired at magnifications of 100, 250, 500, and 1000×. Sample preparation consisted of cutting a section across the width of the molded specimen. The cut was obtained from the central region of the specimen, and then the section was embedded in epoxy resin and placed inside a mold. Once the epoxy resin hardened, it was polished with sandpaper (No. 1000 and 1200) and with alumina powder (particle size of 1 micrometer). The polished surface was chemically etched for 20 min with a solution of sulfuric acid and 7% potassium permanganate. Then, it was rinsed with distilled water and stored in a desiccator. To avoid charging effects during the observation in the SEM, a thin layer of gold was deposited on the prepared samples by a sputtering operated at 40 mA for 40 s.

### 2.5. Statistical Analysis of Results

An individual two-way analysis of variance (ANOVA) was performed for quantifying the effect of the experimental variables (melt and mold temperatures) on the different response variables (spherulites size and crystallinity degree). The surface response methodology (SRM) was employed to visualize the influence of the experimental factors and corresponding levels on the response variables. Equation (1) was employed for establishing quantitative approaches derived from the SRM results. These response functions relate the effect of the two independent variables (X_i_ and X_j_) on the specific response variable (Y) as:

Y = b_0_ + b_i_X_i_ + b_j_X_j_ + b_ii_X_i_^2^ + b_jj_X_j_^2^ + b_ij_X_i_X_j_ (1)

where Y is the predicted response variable, X_i_ and X_j_ are the input variables, X_i_^2^ and X_j_^2^ are the square effects, and X_i_X_j_ is the interaction effect. The b_x_ are regression terms obtained after fitting the curve: b_0_ is the offset term, b_i_ and b_j_ are the linear effects, b_ii_ and b_jj_ are the squared effects, and b_ij_ is the interaction effect.

## 3. Results and Discussion

### 3.1. Spherulite Size Distribution

**Figure 1:**
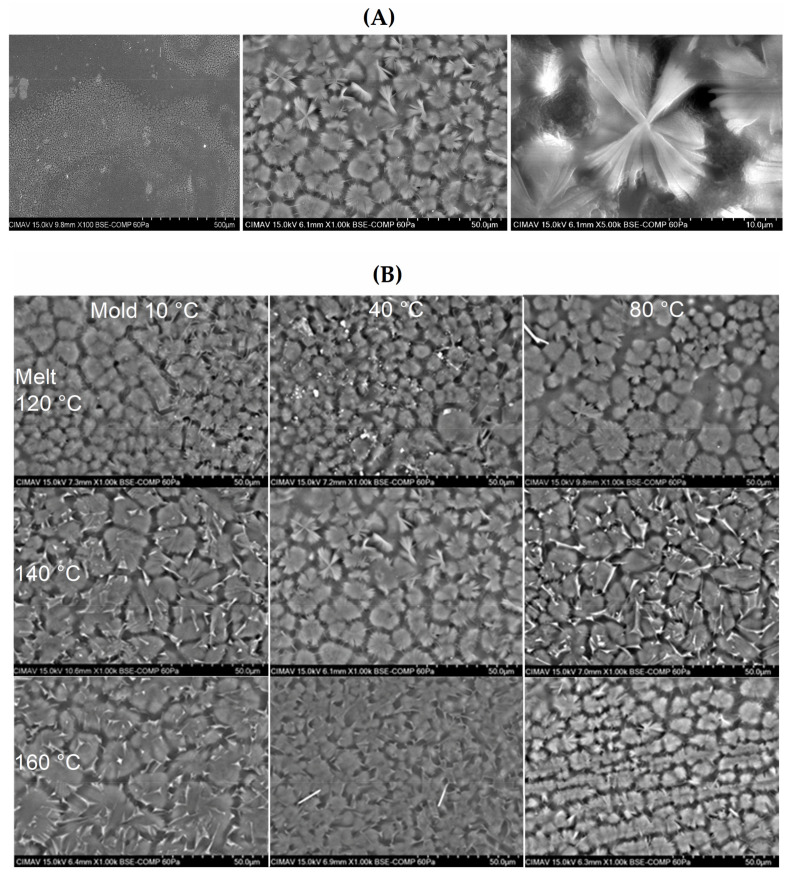
(**A**) Micrographs at different magnifications of the surface of a sample where spherulites were found. (**B**) Representative micrographs at 1000× of the spherulites of LDPE injection-molded specimens.

**Figure 2:**
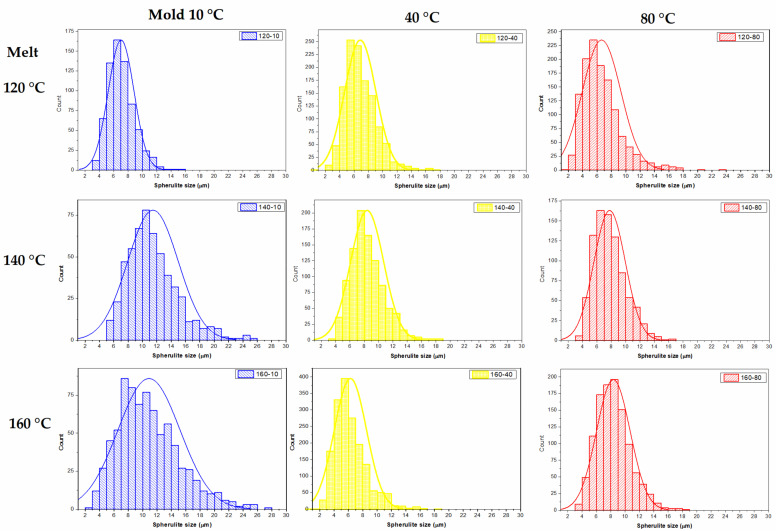
Histogram for the spherulite size distribution of LDPE injection-molded specimens.

Figure 1 shows representative micrographs for each of the samples molded at the defined conditions. Spherulites were observed in different zones of the prepared specimens (Figure 1A). At low magnification (100×), spherulites were observed as a discontinuous grain structure, surrounded by continuous regions where the amorphous polymer remained after the etching. At higher magnifications (1000× and 5000×), the spherulites were observed as hemispherical particles with radially grown lamellae. The empty area between adjacent spherulites corresponds to the removed amorphous polymer during etching. Other features observed in the micrographs were: (i) A thin layer of polymer causing an effect of an under-focused image. (ii) Dissolved spherulites, presented as surface fragments of the lamella. (iii) Deposited gold, observed as high contrast rods or irregular shape particles. However, other morphologies, such as band rings and shish kebabs found in the bulk of gas-assisted injection-molded LDPE specimens, were not observed [3]. The average size of the spherulite was determined by measuring the length of at least 600 spherulites on micrographs acquired at 1000× for each sample (Figure 1B). Figure 2 shows the corresponding histogram for the spherulite size distribution of injection-molded LDPE specimens. Spherulite size distribution tends to displace toward larger particle size with melt temperature, while the amplitude of the distribution becomes narrow with the increase in the mold temperature. These observations were confirmed by numerically comparing the average spherulite size and standard deviation reported in Table 2. The larger average size was observed for samples prepared at the lower mold temperature (10 °C), and spherulite size decreased with the increment in this temperature. The increase in the barrel temperature also increased the size of the spherulite. Overall, spherulite size was relatively smaller (6–11 μm) than those reported in the literature for compression-molded LDPE (10–14 μm) [10].

According to Liparoti et al. [26] for iPP, the microstructural organization typically found in the thickness of injection-molded specimens is known as skin-core morphology and comprises four distinct regions, named: (i) Oriented skin layer composed of globular elements. (ii) Highly oriented non-spherulitic zone or shear layer. (iii) Transitional or morphology evolving region. (iv) Spherulitic core with small orientation. Each of these regions differs in morphology and properties, such as elastic modulus. In addition, they observed that as the mold temperature increases, the cooling rate decreases, providing enough time for the microstructural arrangement of the core region. According to Kamal and Chu [40], HDPE containing a nucleating agent developed a grainy microstructure composed of small spherulites (about 10 μm) that remained unchanged with the temperature conditions in isothermal and non-isothermal crystallization. However, when HDPE without a nucleating agent was molded, the polymer developed a microstructure composed of a transcrystalline region and spherulites (about 100 μm) deformed in the direction of the heat removal. Another factor influencing the microstructure is the thickness of the molded specimen. For specimens thicker than 100 nm, spherulites are formed in the bulk by a surface crystallization process [11]. The samples analyzed herein were cut from the cross-section perpendicular to the injection flow, and the micrographs shown are representative of the sample and were acquired from different areas of the sample. As mentioned above, no other morphologies like those described in [26] were observed in the samples. This indicated that the supramolecular structure of LDPE of injection-molded specimens was spherulitic.

Although the spherulites may deform and elongate in the direction of the flow under intermediate levels of deformation [36], the slight deformation observed in some spherulites was caused by the mechanical deformation exerted during the sample preparation, i.e., polishing.

### 3.2. Qualitative Characterization

**Figure 3:**
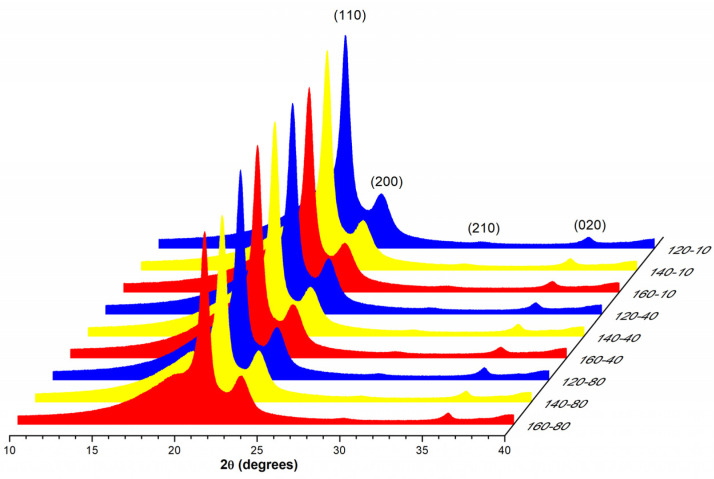
XRD diffractograms of LDPE injection- molded specimens.

Figure 3 shows the XRD diffractograms of LDPE injection-molded specimens. The most distinguishable features are the diffraction peaks corresponding to the different crystalline planes in LDPE at 2θ of 21.3, 23.6, 29.7, and 36.1°, and the broad peak located at 19.3° associated with the amorphous segments of polymer. The visual comparison shows little changes in the main crystalline plane (110) intensity, suggesting the difficulty of deducing qualitative relations between the experimental conditions and the intensity values from XRD diffractograms. For example, the ratio of amorphous to crystalline intensities (Table 2) showed similar values about 0.26 for each experimental condition. In addition, Zheng et al. [6] reported a value of 0.22 for an LDPE sample rapidly solidified between a polyethylene terephthalate (PET) film by compression molding and a value of 0.21 when solidification was slower.

In diffraction, the intensity is related to the number of diffracting planes, while the integration of the area under the curve is related to the diffracting volume and phase fraction. Thus, the area under the curve was employed for determining the crystallinity degree (X). This parameter was calculated by dividing the area of the diffracting peaks by the total area of the amorphous and crystalline peaks. The calculated values expressed in percentage (%) are presented in Table 2. The crystallinity degree showed values in the range of 29.8–33.9%. These values were relatively lower than those calculated from DSC measurements [8] because the latter are usually obtained by normalizing the melting enthalpy with a constant value of a fully crystallized sample. Liparoti et al. [41] analyzed by XRD the heated side of iPP samples molded by asymmetric heating. They found a crystallinity degree in the range of 55–61% and explained that the differences in the crystallinity are caused by the orientation index, described as the ratio of the diffraction peaks. According to them, the orientation of the microstructure decreases with the increase in the mold temperature because there is more time to relax the molecular stretch. This suggested that the differences in the intensity of the (110) diffraction peak may be caused by different orientations rather than by slight variations in the crystallinity.

In isothermal crystallization, increasing the temperature of the mold or the crystallization temperature induces a lower degree of crystallinity in the sample [40,42]. At these conditions, the nucleation and growth process of crystals, such as spherulites, are slower. This means that longer crystallization times will be required to reach a certain value of the degree of crystallinity. On the other hand, in non-isothermal crystallization, a slow cooling rate promotes a higher degree of crystallinity. However, there is also evidence showing an opposite behavior—that is, a higher cooling rate induces a higher degree of crystallinity [21]. Nevertheless, from these two thermal processes, injection molding is governed by non-isothermal crystallization, where the cooling rate mainly depends on the logarithmic ratio of the injection nozzle and mold temperatures. The cooling rate increases with the temperature of the nozzle, while it decreases with the temperature of the mold. Recent Large-Scale Atomic/Molecular Massively Parallel Simulations (LAMMPS) have shown that the increase in crystallinity is affected by the release of heat and changes in the density of the polymer [43]. Likewise, the experimental conditions, such as the temperature difference between the molten polymer and the mold, the injection pressure, and the high molecular weight, strongly favor the crystallization of LDPE. In addition, these simulations showed that the crystallinity degree rapidly increases about 20% during the first microsecond of quenching. The results presented herein suggest that the crystallinity of the LDPE was relatively stable over the range of selected molding temperatures, reaching similar values in the first seconds of the solidification within the mold.

### 3.3. Quantitative Analysis by ANOVA and SRM

ANOVA is used to determine the quality of the data, i.e., if the means of the different groups are significantly similar or not, and to quantify the contribution of each of the factors [30]. The F-test is employed for comparing the experimental factors. If the F-value is equal to or lower than 1 (F < 1), the effect of the factor is null. While if F > 1, the factor influences the response variable. In order to test the quality of the data, the probability (*p*-value) must be lower than the significance level (0.05).

The calculated ANOVA for the spherulite size and the crystallinity percentage are reported in Table 3. Slight differences were observed between the ANOVA for the two response variables. For the spherulite size, both factors (melt and mold temperatures) were significant on the response variable. However, the effect of the mold temperature was about 1.32 times higher than the melt temperature. The individual factors, melt and mold temperatures, were 2.4 and 3.2 times higher than the interaction factor, respectively.

The calculated ANOVA for the crystallinity of the injected-molded specimens showed that the effects of the two factors and their interaction were significant (*p*-value). However, the quantification of the effect (F-value) was relatively smaller. For example, the effect of the mold temperature was 3.4 times higher than the melt temperature. Conversely, the individual factors, melt and mold temperatures, were 1.3 and 4.4 times higher than the interaction factor, respectively.

**Figure 4:**
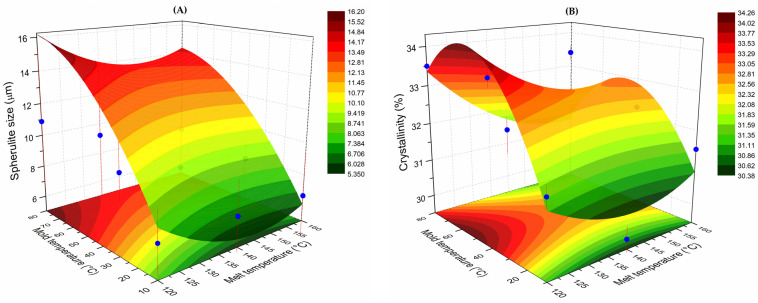
Surface response plots (SRP) for (**A**) spherulite size and (**B**) crystallinity.

Figure 4 shows the response surface plots (SRP) for the spherulite size and crystallinity. The behavior of the spherulite size was close to what was expected, i.e., the largest sizes were obtained at the hottest molding conditions (melt 160 °C and mold 80 °C). Under these conditions, the molten polymer chains have enough time to arrange in a more orderly way, allowing the lamellae to grow, being observed as larger spherulites. On the other hand, at the coldest molding conditions (melt at 120 °C and mold at 10 °C), solidification is carried out more rapidly, providing less time for the nucleation and growth of the spherulites. The color fringes of the SRP indicated an almost linear behavior of the size of the spherulite with the mold conditions, where the increase in the temperature of the mold influenced the increase in the size. For example, for a spherulite size of 10 μm (yellow fringe), the size hardly varies over the range of melt temperatures.

Derived from the non-linear surface fit, the predicting model for the spherulite size is presented in Equation (2):

S = 81.91 + 0.2428X − 1.08022Y − 0.00137X^2^ + 0.00377Y^2^ − 6.83 × 10^−4^XY (2)

where S is the predicted spherulite size, X is the mold temperature, and Y is the melt temperature. The coefficient of determination (R^2^) from the SRP fit was 0.789. As observed from the equation, the mold temperature presented a low positive contribution (0.2428), while the contribution of the melt temperature was negative (−1.108022). The contribution of the square factors was in the same order of magnitude but with contrary signs, i.e., negative for the mold and positive for the melt. The contribution of the interaction of the two factors (XY) was negative.

The shape of the SRP for the crystallinity was different from that of spherulite size. The highest crystallinity values (orange to dark red fringes) were obtained at intermediate molding conditions, i.e., low melt temperatures (120 °C) and high mold temperatures (>40 °C). In contrast, the lowest crystallinity values (green to yellow fringes) were obtained in two opposite areas of the surface: (i) At low mold temperatures over all the range of melt temperatures; (ii) At high mold temperatures (80 °C) and intermediate and high melt temperatures (>140 °C). The predicting model for the crystallinity is presented in Equation (3):

C = 59.97 + 0.2412X − 0.4424Y − 0.00149X^2^ + 0.00158Y^2^ − 6.29 × 10^−4^XY (3)

where C is the predicted crystallinity (%), X is the mold temperature, and Y is the melt temperature. The coefficient of determination (R^2^) from the SRP fit was 0.451. The contribution of the mold temperature was positive (0.2412), while that of the melt temperature was negative (−0.4424). The square factors were in the same order of magnitude but differed in the sign, negative for the mold temperature and positive for the melt temperature. The contribution of the two factors was negative. It is worth mentioning that the SRPs and the derived predicting models are only valid in the molding conditions employed in this work, i.e., 120–160 °C for the melt temperature and 10–80 °C for the mold temperature.

In order to understand the differences in the shape of the two response surfaces, it is necessary to explain the way the characterizations were performed. Although both characterizations involve the analysis of injection-molded specimens, the measurement of the size of the spherulites was carried out in the cross-section of the sample by devastating during the polishing a few hundred micrometers from the surface and removing with the acid solution the amorphous phase by etching. On the other hand, the X-ray analysis was carried out on one of the sides of the specimen. In this case, the penetration of the X-ray beam is in the order of tens of micrometers, and its path through the sample volume involves the interaction with both crystalline and amorphous phases. Additionally, the ANOVA was calculated with a set of 548 data per experimental condition, while the XRD calculation involved two measurements per sample. Evidently, these facts may give rise to different behaviors in both the ANOVA and the RSP.

Based on the melting enthalpy of LDPE, Leyva-Porras et al. [8] observed an increase in the degree of crystallinity with the mold temperature and a decrease with the melt temperature. They qualitatively explained that in injection-molded samples, the increase in crystallinity was caused by the increase in the number of nuclei sites that subsequently grew as spherulites. According to Hall, Percec, and Klein [43], the high pressure exerted during the injection-molding process acts as a large driving force to induce long-chain crystallization (nucleation). In the present work, it was quantitatively demonstrated that the interaction of the two processing variables (melt and mold temperatures) largely influenced the development of the microstructure in LDPE processed at high shear rates. Clearly, the processing condition exerted herein promoted the nucleation and growth of crystalline moieties, observed as the variations in the spherulite size and changes in the crystallinity.

## 4. Conclusions

The effect of the processing conditions of injection-molded LDPE specimens on the microstructure and crystallinity was studied. The variations in the melt and mold temperatures induced an average spherulite size of 6–11 μm, while the degree of crystallinity was 29.9–33.9%. These results suggested the relatively high stability of the LDPE to modify its microstructure over the range of exerted molding temperatures. A two-way ANOVA was employed to quantify the effect of the molding conditions. The factor with the major effect on the spherulite size and crystallinity was the mold temperature, with less effect the melt temperature, while the interaction of the ywo variables presented the lowest effect. SRPs were constructed employing a second-order model that was easy to relate to the physical phenomenon. The spherulite size increased at high mold temperatures and remained almost invariable in all the range of melt temperatures. The behavior of the crystallinity presented higher values at intermediate molding conditions and lower values at two processing conditions. A predicting model was derived from each data set. In both models, the mold temperature presented a positive effect; the melt temperature presented a negative effect, while the interaction of the two factors was also negative but numerically significant. The results presented herein are valuable for setting empirical relations between the microstructure, crystallinity, and the molding conditions of LDPE.

## Figures and Tables

**Table 1:** Processing conditions for the injection molding of LDPE specimens.

Processing Condition	Value
Barrel rotation speed	120 (RPM)
Back pressure	5 (bar)
Filling pressure	100 (bar)
Cooling time	60 (s)

**Table 2:** Summary of the spherulite size and crystallinity measurements of LDPE injection-molded specimens.

Injection Molding Condition (Melt–Mold)	Spherulite		Crystallinity	
	Average Size (μm)	Std. Dev. (μm)	Ratio (A/C)	X (%)
120–10	7.03	1.80	0.26	31.5
120–40	6.87	2.17	0.26	29.8
120–80	6.64	2.68	0.27	31.5
140–10	11.46	3.53	0.27	33.6
140–40	8.39	2.30	0.26	33.9
140–80	7.76	2.18	0.26	32.2
160–10	10.88	4.32	0.26	33.5
160–40	6.28	2.25	0.26	31.4
160–80	8.35	2.34	0.28	31.7

**Table 3:** ANOVA results determined for the spherulite size and crystallinity degree of LDPE injected-molded specimens.

	Spherulite Size					Crystallinity				
Source	DF ^a^	SS ^b^	MS ^c^	F	P ^d^	DF ^a^	SS ^b^	MS ^c^	F	P ^d^
**Melt**	2	5126.83	2563.41	351.23	0	2	4.89 × 10^−4^	2.41^−4^	21.22	3.91^−4^
**Mold**	2	6798.29	3399.14	465.74	0	2	0.00167	8.32^−4^	73.04	2.73^−6^
**Interaction**	4	4203.62	1050.9	143.99	0	4	7.54^−4^	1.88^−4^	16.53	3.53^−4^
**Model**	8	16,128.75	2016.09	276.24	0	8	0.0029	3.62^−4^	31.83	1.02^−5^
**Error**	4923	35,929.47	7.29			9	1.02^−4^	1.14^−5^		
**Total**	4931	52,058.23				17	0.00301			

^a^ Degrees of freedom. ^b^ Sum of squares. ^c^ Mean squares. ^d^ Calculated at a significance level of 0.05.

## Data Availability

Data are contained within the article.
